# Detection and Prevalence of Rabies in Bats from Oaxaca

**DOI:** 10.3390/microorganisms13061417

**Published:** 2025-06-18

**Authors:** María Isabel Medina Matías, Margarita García-Luis, Oscar Ezequiel Blanco Esquivel, Israel Nicolás Reyes, Miguel Ángel Domínguez Martínez, Gisela Fuentes-Mascorro

**Affiliations:** 1 Escuela de Ciencias, Universidad Autónoma Benito Juárez de Oaxaca, Av. Universidad s/n Ex Hacienda Cinco Señores, Oaxaca CP 68120, Mexico; iisa.bio.bat01@gmail.com; 2 Laboratorio de Investigación en Salud Ecosistémica (LInSE), Cuerpo Académico Ciencias Veterinarias Aplicadas al Desarrollo Regional, Universidad Autónoma Benito Juárez de Oaxaca, Av. Universidad s/n Ex Hacienda Cinco Señores, Oaxaca CP 68120, Mexico; 3 Departamento de Zoología, Pabellón Nacional de la Biodiversidad-Instituto de Biología, Universidad Nacional Autónoma de México, Circuito Centro Cultural, Ciudad Universitaria, Ciudad de México CP 04510, Mexico; 4 Universidad para el Bienestar Benito Juárez de Oaxaca, Av. Universidad, Colonia Loma del Zacate, Concepción del Progreso, Putla Villa de Guerrero CP 71001, Mexico; oscarbco@hotmail.com; 5 Centro Nacional de Servicios de Diagnóstico en Salud Animal (CENASA), Avenida Centenario de la Educación s/n (k/m 37.5 Carretera Federal México-Pachuca), Tecámac CP 55740, Mexico; israel.nicolas@senasica.gob.mx; 6 Laboratorio de Genética Molecular y Zoonosis, Cuerpo Académico Ciencias Veterinarias Aplicadas al Desarrollo Regional, Universidad Autónoma Benito Juárez de Oaxaca, Av. Universidad s/n Ex Hacienda Cinco Señores, Oaxaca CP 68120, Mexico; madm02@hotmail.com; 7 Laboratorio de Investigación en Reproducción Animal (LIRA), Cuerpo Académico Ciencias Veterinarias Aplicadas al Desarrollo Regional, Universidad Autónoma Benito Juárez de Oaxaca, Av. Universidad s/n Ex Hacienda Cinco Señores, Oaxaca CP 68120, Mexico; lirauabjo@gmail.com

**Keywords:** direct immunofluorescence (DIF), Lyssavirus, livestock, rabies virus, zoonosis

## Abstract

The rabies virus (genus Lyssavirus), is a deadly zoonotic agent affecting humans and animals. Although Mexico has been declared free of canine rabies (V1), sylvatic rabies persists. This study aimed to determine the prevalence of the virus in *Desmodus rotundus* and other non-hematophagous bat species in Oaxaca. The methodology comprised four stages: a literature review, data requests to the Servicio Nacional de Sanidad, Inocuidad y Calidad Agroalimentaria (SENASICA), fieldwork using mist nets across 15 municipalities in Oaxaca, and diagnosis via direct immunofluorescence at the Centro Nacional de Servicios de Diagnóstico en Salud Animal (CENASA). SENASICA reported 89 positive rabies cases (2014–2023) across six laboratories, with the majority (67.02%) attributed to the Oaxaca State Public Health Laboratory. Among the 194 bats analyzed (129 *D. rotundus*), only three tested positive for the virus, yielding a prevalence of 1.54%. Positive cases were exclusively identified in *D. rotundus* from San Lucas Ojitlán and The Heroic City of Tlaxiaco. This prevalence aligns with that of national studies, which ranges from 0.05% to 3%. These findings underscore the need to maintain epidemiological surveillance in wild and domestic fauna, alongside public awareness campaigns highlighting bats’ ecological importance for ecosystem conservation and the risks associated with their decline.

## 1. Introduction

Mexico has 144 bat species [1] and ranks fourth among countries with the highest number of endemic species (13 species) [2]. Oaxaca exhibits remarkable bat diversity, with 96 of the 144 species found nationwide [3]. However, in recent decades, many bat species have experienced a drastic population decline, with over 21% of microchiropterans classified as threatened and another 23% considered at risk [4]. Fragmentation, deforestation, the widespread misconception that all bat species are vampire bats—leading to human persecution [5]—and land-use changes have negatively impacted the abundance and diversity of all taxonomic groups [6,7].

Bats are suitable reservoirs for viruses and other pathogens due to their unique biological and ecological characteristics, such as colonial behavior, flight capability, seasonal migration, daily movement patterns, the ability to enter torpor and hibernation, and a relatively long lifespan compared to other mammals of similar size. For example, species that form large population densities during roosting increase the likelihood of intra- and interspecific viral transmission [8,9].

Rabies is one of the most extensively studied bat-borne diseases due to the economic losses it causes in the livestock sector. Since the late 20th century, Mexico has implemented control campaigns to control hematophagous species and determine rabies in samples sent to several laboratories nationwide. Mexico is considered free of canine rabies [10], thanks to sustained dog vaccination campaigns and the national epidemiological surveillance system. However, wildlife rabies remains a public health concern, as the virus persists among the wide range of wild reservoirs present in nature [11]. According to reports from the National Epidemiological Surveillance System of the Dirección General de Salud Animal (SENASICA), a total of 310 positive cases of paralytic bovine rabies were reported nationwide between January and December 2024 [12].

From 1993 to 2024, Mexico documented approximately 216 human rabies cases, 31.5% of which were attributed to dog bites [13,14,15]. However, sylvatic transmission also accounted for numerous cases. Between 1993 and 2002, 56 human cases were associated with wildlife, primarily bats (44 cases), followed by skunks, bobcats, coyotes, and a fox [13]. These infections typically occurred in rural areas with limited access to medical services [11].

Currently, 1895 municipalities in Mexico are considered under “rabies control”, while 568 are classified as “naturally free” due to the absence of environmental conditions for rabies reservoir species [16]. In 2024, 563 rabies diagnostic reports were issued, of which 55.1% tested positive. The majority of these occurred in livestock, especially cattle, but hematophagous bats were also confirmed as vectors [12].

Globally, rabies remains a major zoonotic disease with an estimated 59,000 annual human deaths, mainly in developing countries [17]. While significant advances have been made in dog rabies control, sylvatic rabies in bats remains a challenge in Latin America [17,18]. In Mexico, bat rabies is especially relevant in states such as Oaxaca, where endemic circulation of the virus has been reported. Historical records indicate over 51 bovine rabies cases in the state between 2020 and 2024 [12], and more recent reports continue to document virus activity.

The Direct Immunofluorescence (DIF) test remains the gold standard recommended by the World Organization for Animal Health (WOAH) for rabies diagnosis, particularly in field conditions [19]. Recent studies in Mexico and South America confirm its continued use in surveillance and epidemiological investigations [20,21,22]. Given this context, the aim of this study was to determine the presence and prevalence of rabies virus in *D. rotundus* and other non-hematophagous bat species captured through active surveillance in various municipalities of Oaxaca. This effort supports disease prevention within a One Health framework and contributes to public health policy and bat conservation.

## 2. Materials and Methods

**Figure 1:**
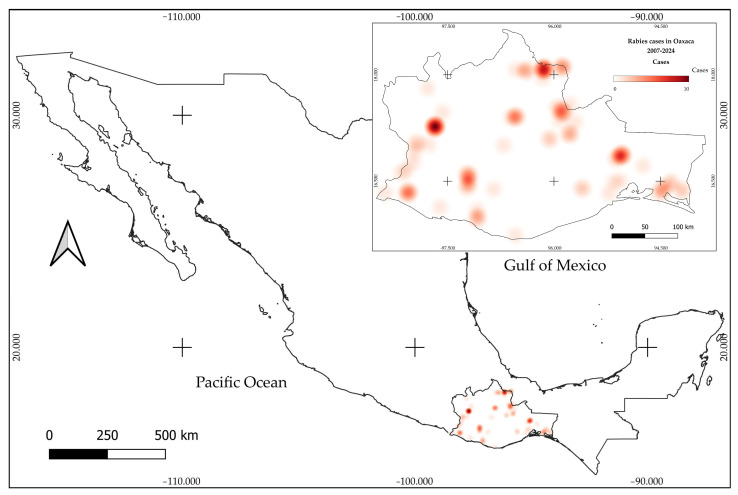
Location of the state of Oaxaca within Mexico and spatial distribution of rabies cases by physiographic province. A color gradient indicates the intensity of reported cases, with darker tones representing provinces with higher cumulative incidence from 2007 to 2024.

The study was conducted in Oaxaca, located in the southern portion of the Mexican Republic. It borders Veracruz and Puebla to the north and northeast, Chiapas to the east, the Pacific Ocean to the south, and Guerrero to the west. Its geographical coordinates range from 15°39′ to 18°39′ N and 93°52′ to 98°32′ W. Oaxaca covers an area of 95,364 km^2^, representing 4.8% of the national territory [23]. A total of 26 vegetation types have been described, corresponding to nine physiognomic-floristic provinces [24]. Due to its geographical position, complex topography, and exposure to meteorological systems from both the Pacific and Gulf of Mexico slopes, the state presents contrasting thermal and humidity conditions [25] (Figure 1).

### 2.1. Desk-Research

To identify geographic locations with confirmed rabies cases in Oaxaca, a comprehensive search was conducted using keywords in both English and Spanish: rabies, bats, hematophagous, *Lyssavirus*, direct immunofluorescence, and RABV. Searches were carried out across scientific databases, including BioOne, Scopus, Web of Science, Redalyc, and Scielo, as well as in thesis repositories from national and international institutions and on websites of the World Health Organization (WHO), the General Directorate of Epidemiology (DGE), and PAN (National Overview of Paralytic Bovine Rabies). In addition, historical case information was requested from SENASICA (National Service for Agri-Food Health, Safety, and Quality), CENASA (National Center for Animal Health Diagnostic Services), and CENAPRECE (National Center for Preventive Programs and Disease Control).

### 2.2. Fieldwork (Sample Collection)

Captures of both hematophagous and non-hematophagous bat species were carried out in Oaxaca between 2022 and January 2024. Bat captures were conducted in 15 municipalities; six of these were selected based on ecological conditions suitable for the presence of *Desmodus rotundus*, despite no prior reports of hematophagous bat bites to CEFPPO (Comité Estatal para el Fomento y Protección Pecuaria del Estado de Oaxaca). The remaining sites were chosen from areas where rabies-positive cases in livestock had been previously reported. This was conducted under collection permit 20/k5-0025/10722, issued by the Secretaría de Medio Ambiente y Recursos Naturales (SEMARNAT). Specimens were captured in caves, mines, and livestock enclosures. Capture efforts were scheduled according to the lunar calendar, with mist nets (nylon thread, 2.5 m wide, 12 m long) set up around corrals and near caves during nighttime hours.

Nets were checked every 30 min to detect any captured bats. When a specimen was found, it was carefully removed from the net. Each bat was identified at the species level based on morphological characteristics and feeding habits (e.g., size, snout shape, hematophagous vs. non-hematophagous), using the dichotomous key Bats of Mexico [26].

Euthanasia was then performed following the Euthanasia Reference Manual [27] and the Manual of Techniques for Capturing, Preparing, Preserving, and Studying Specimens, employing the asphyxiation method (for very small mammals <30 g), using pressure to the cardiopulmonary region or cervical dislocation. The latter is the most commonly used method due to its speed and minimal pain [28]. Collected specimens were placed in coolers and transported to the Animal Reproduction Research Laboratory (LIRA), where they were stored at −20 °C. Collection site coordinates were recorded using a GPS device.

### 2.3. Brain Extraction

All specimens were processed inside a Class IIA biosafety cabinet. Forceps were used to hold the specimen in place and prevent direct contact. Dissection scissors were used to remove part of the skin and hair from the head, followed by a transverse cut through the entire brain. The brain was then extracted and placed into cryotubes, which were labeled with the corresponding identification data for each specimen [19,29].

### 2.4. Preparation of Imprints

A small piece of brain tissue was placed onto a microscope slide using a wooden stick. The slide was then pressed against a sheet of paper to create a thin tissue smear. The impressions needed to be very thin; otherwise, nonspecific fluorescence would increase. Two imprints per specimen were prepared on each slide and labeled according to the corresponding specimen processed [19,29].

### 2.5. Direct Immunofluorescence Technique (DIF)

The direct immunofluorescence technique was conducted at CENASA following WOAH [19] and NOM ZOO-056-ZOO-1995 standards [29]. Brain smears were prepared by placing a thin tissue impression on microscope slides using a wooden stick. The slides were fixed in 100% acetone at −20 °C for 30 min to enhance tissue adhesion and permeability. After air-drying, 50 µL of FITC (isocyanate fluorescein, which binds to the rabies antigen and emits fluorescence under the microscope, IgG2a isotype)-labeled anti-rabies monoclonal globulin (conjugate) was applied to each imprint. Slides were incubated at 37 °C in a humid chamber for 30 min, washed with PBS and distilled water, and mounted with phosphate-buffered glycerin under coverslips.

Each slide included a positive control (CVS, Challenge Virus Standard, in mouse brain tissue with high fluorescence intensity ++++, established using the CVS-11 strain, which was characterized by CENASA through whole-genome sequencing, real-time PCR, and immunofluorescence testing) and a negative control (mouse brain tissue without rabies virus presence; its metrological traceability is established by CENASA through real-time PCR and immunofluorescence testing to confirm the absence of the rabies virus) to ensure assay validity. Two brain imprints per specimen were examined under an HBO100 fluorescence microscope (Carl Zeiss, Oberkochen, Germany) at 400× magnification. For each sample, a minimum of three microscopic fields were analyzed to confirm the presence of specific apple-green fluorescence. This signal results from the binding of the FITC-labeled anti-rabies monoclonal antibody (IgG2a isotype) to viral antigens present in tissue infected with the CVS-11 strain. Fluorescence intensity was graded from negative to 4+, in accordance with NOM ZOO-056-ZOO-1995 and WOAH diagnostic criteria, reflecting the relative abundance of antigen [19,29].

### 2.6. DIF Microscopic Observation and Interpretation of Results

Slides were examined using an HBO100 fluorescence microscope, beginning with the positive control to identify the characteristics of specific fluorescence. The negative control was then observed to detect nonspecific fluorescence, followed by the test samples [19,29]. According to the Mexican Official Standard NOM-056-ZOO-1995, fluorescence intensity ranges from negative to 4+ and is directly proportional to the specific antigen present. Rabies virus is identified by its characteristic apple-green fluorescence.

### 2.7. Rabies Prevalence

Overall prevalence was calculated using the following formula [30]:

%P = (Number of positive individuals/Total number of individuals) × 100

Based on this, the following formulas were applied to calculate prevalence at different scales within the study:

%Prevalence by species = (Number of positive individuals of the species/Total number of individuals of the species) × 100

%Prevalence by municipality = (Number of positive individuals in the municipality/Total number of individuals in the municipality) × 100

%Prevalence by region = (Number of positive individuals in the region/Total number of individuals in the region) × 100

## 3. Results

The results are presented in three main parts: historical records of rabies cases in Oaxaca, findings from the active bat surveillance conducted during 2022–2024, and rabies prevalence detected through direct immunofluorescence. Together, these findings provide information on the historical and current rabies circulation in the state of Oaxaca.

### 3.1. Historic Records

**Figure 2:**
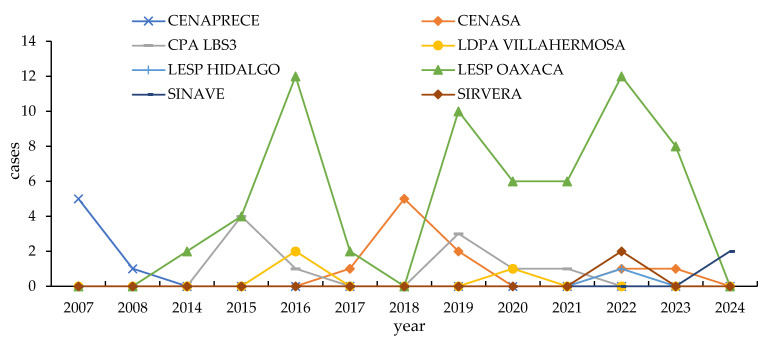
Records of rabies-positive cases based on DIF diagnoses, as reported by SENASICA (Servicio Nacional de Sanidad, Inocuidad y Calidad Agroalimentaria) and CENAPRECE (Centro Nacional de Programas Preventivos y Control de Enfermedades) in the state of Oaxaca from 2007 to January 2023. CENASA, Centro Nacional de Servicios de Diagnóstico en Salud Animal; LDPA Villahermosa, Laboratorio De Patología Animal Villahermosa; CPA LBS3s, Laboratorio de Bioseguridad Nivel 3 de la Comisión México-Estados Unidos para la Prevención de Fiebre Aftosa; LESP, Laboratorio Estatal de Salud Pública; SINAVE, Sistema Nacional de Vigilancia Epidemiológica; SIRVERA, Sistema de Información Regional para la Vigilancia Epidemiológica de la Rabia.

According to epidemiological data from CENASA, the majority of rabies cases in cattle in Oaxaca are attributed to transmission by hematophagous bats, specifically *Desmodus rotundus*. No other wildlife species were implicated during the study period. CENASA reported 86 records from six laboratories in Oaxaca from 2014 to 2023, including confirmed rabies cases in dogs, cattle, bats, and humans. The laboratory with the highest number of records was the Oaxaca State Public Health Laboratory (LESP Oaxaca), accounting for 70.8% (63 records) of the total, followed by the Biosafety Level 3 Laboratory of the Mexico–United States Commission for the Prevention of Foot-and-Mouth Disease (CPA LBS3), with 13.5% (12 records). The laboratories with the fewest records were LESP Hidalgo and the Animal Pathology Laboratory in Villahermosa, Tabasco (LDPA Villahermosa), with one and three cases, respectively. The years with the highest number of confirmed cases were 2016 and 2019, each with 15 cases, followed by 2018 with 10 cases. The CENAPRECE data do not specify the laboratory where the analysis was conducted, but they report five positive rabies cases in dogs—four in 2007 and one in 2008—as well as one human case in 2007. According to SIRVERA system data, two human cases were reported in 2022, and SINAVE data indicate two additional human cases in 2024. In total, 99 rabies-positive cases were documented in Oaxaca between 2007 and 2024 (Figure 2).

**Figure 3:**
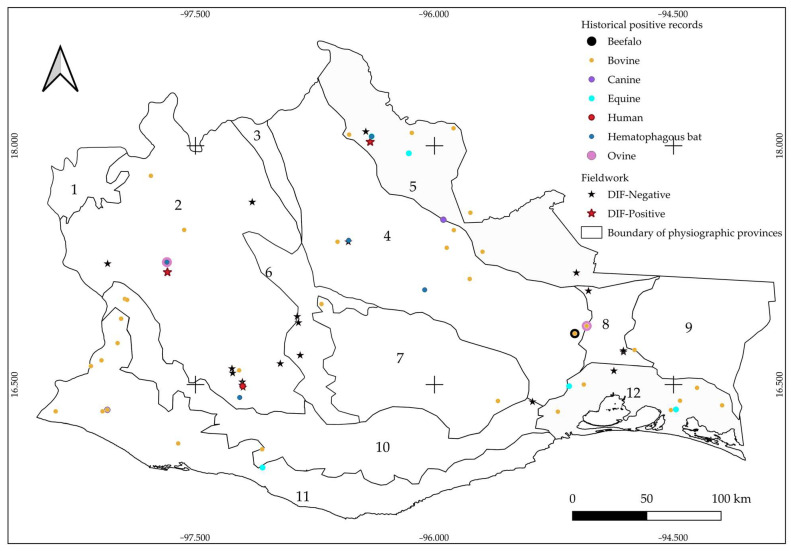
Distribution of historical records obtained from CENASA, CENAPRECE, SINAVE, and SIRVERA and sampling sites from the present study. Colored circles represent historical records of rabies-positive cases from 2007 to 2024. Black stars indicate sampling sites with no positive cases found during fieldwork. Red stars represent positive cases identified in the present study. Physiographic provinces: (1) Balsas Depression, (2) Western Mountains and Valleys, (3) Tehuacan Valley, (4) Sierra Madre de Oaxaca, (5) Gulf Coastal Plain, (6) Central Valleys, (7) Central Mountains and Valleys, (8) Isthmus Depression of Tehuantepec, (9) Sierra Madre del Sur of Oaxaca and Chiapas, (10) Sierra Madre del Sur, (11) Pacific Coastal Plain, (12) Tehuantepec Coastal Plain.

These cases were geographically distributed across 47 municipalities (8.2% of the total in the state). The municipality with the highest number of confirmed cases was Heroica Ciudad de Tlaxiaco, accounting for 10.1% (10 cases), followed by San Juan Bautista Tuxtepec with 8.1% (eight cases). Twenty-six municipalities reported only one case each (Appendix A, Table A1). The physiographic provinces with the highest cases were the Gulf Coastal Plain and the Sierra Madre de Oaxaca, each with 19.2% (19 cases). In 2022—with 16 confirmed cases—rabies was reported in the following physiographic provinces: Western Mountains and Valleys, Sierra Madre del Sur, Sierra Madre de Oaxaca, Central Mountains and Valleys, Isthmus Depression, and Gulf Coastal Plain (Figure 3). Two physiographic provinces reported no cases in their municipalities: Balsas Depression and Tehuacan Valley.

By species, cattle had the highest percentage of rabies-positive diagnoses, accounting for 70.7% (70 cases), followed by the vampire bat *Desmodus rotundus*, with 9.1% (9 cases). The physiographic provinces where positive cases of hematophagous bats were recorded included Western Mountains and Valleys, Sierra Madre of Oaxaca, and Gulf Coastal Plain. Meanwhile, Sierra Madre of Oaxaca and the Gulf Coastal Plain reported the highest number of rabies-positive cattle cases, with 15 in each (Appendix A.1).

### 3.2. Active Bats Surveillance (2022–2024)

Bat captures were carried out in 15 municipalities within the state, covering six of Oaxaca’s twelve regions: Western Mountains and Valleys, Central Valleys, Isthmus Depression of Tehuantepec, Sierra Madre del Sur, Sierra Norte, and Gulf Coastal Plain (Figure 3). The highest number of individuals was collected in the Isthmus region, while the lowest was recorded in Central Valleys. A total of 194 bats were captured, of which 129 belonged to the vampire bat *Desmodus rotundus*, representing 66.4% of the total. The remaining 35.5% (65 individuals) belonged to other species (Table 1). Three *D. rotundus* individuals were identified as positive for rabies through DIF, and this is indicated in parentheses in Table 1.

### 3.3. Rabies Virus Detection by Direct Immunofluorescence (DIF)

**Figure 4:**
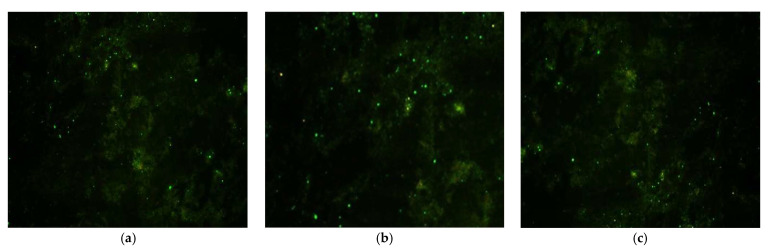
The presence of the rabies virus is evidenced by apple-green fluorescence. Positive DIF cases: (**a**) *Desmodus rotundus* (Heroica Ciudad de Tlaxiaco); (**b**,**c**) *D. rotundus* (San Lucas Ojitlán).

As a result of applying the DIF technique to detect rabies in the captured bats, only three individuals tested positive for the virus (Figure 4). One of these was from the community of Heroica Ciudad de Tlaxiaco, and the other two were from San Lucas Ojitlán (Figure 3). These results were reported to the National Epidemiological Surveillance System (SIVE) to enable appropriate response actions, including cattle vaccination and, most importantly, informing the public about the risks and how to respond if their livestock showed signs of rabies.

### 3.4. Prevalence of Rabies Virus in Bats

The prevalence was calculated at various levels: global, species, municipalities, and regions. Of the 194 bat samples analyzed, three tested positive for rabies, resulting in an overall prevalence of 1.54%. By species, *Desmodus rotundus* was the only one with positive cases, showing a prevalence of 2.3% (3/129), while no cases were detected in the other species, resulting in a prevalence of zero. The site with the highest prevalence was the community of San Lucas Ojitlán, with 33.3% (2/6 individuals), followed by Heroica Ciudad de Tlaxiaco, with 7.6% (1/13). All other municipalities had a prevalence of zero. Among regions, Gulf Coastal Plain showed the highest prevalence at 33.3% (2/6), followed by Western Mountains and Valleys with 1.7% (1/57). The remaining areas showed no positive cases.

## 4. Discussion

The historical records of rabies in Oaxaca reveal that the municipalities most affected are Heroica Ciudad de Tlaxiaco and San Juan Bautista Tuxtepec. At the physiographic province level, the Sierra Madre de Oaxaca and the Gulf Coastal Plain reported the most cases. However, these data may be underestimated, as Oaxaca is a state where communities do not routinely report this issue, primarily due to a lack of information or prevailing beliefs about rabies and bats. Additionally, Oaxaca’s complex topography poses challenges for accessing many of its municipalities. The state also has significant linguistic and cultural diversity, which creates barriers to effectively delivering information. As in other parts of the country, cattle are the most affected species among domestic/agricultural mammals by this disease in Oaxaca.

Despite ongoing rabies surveillance efforts in Mexico, published studies on prevalence in hematophagous bats remain limited. In this study, an overall prevalence of 1.5% (3/194) was recorded—an estimate that falls within the range reported in other states, such as Colima with 0.05% and Guerrero with 3% [31,32]. In La Huasteca Potosina and the state of Sinaloa, a 1.8% prevalence in hematophagous bats has been reported, which aligns with the results of the present study [33]. Our estimate falls well within this expected range, providing further evidence of the low but persistent circulation of the rabies virus in hematophagous bat populations.

Importantly, all three rabies-positive cases in this study were identified in *Desmodus rotundus*, confirming its status as the primary vector of sylvatic rabies in Mexico. The species-specific prevalence in *D. rotundus* was 2.3%, with positive cases detected in two municipalities. In Tlaxiaco, the presence of a positive case corresponds with prior confirmed outbreaks in livestock. In contrast, the cases identified in San Lucas Ojitlán occurred in the absence of recent reports, suggesting undetected viral circulation. These findings emphasize the utility of active surveillance in revealing silent or emerging foci of infection.

No rabies cases were detected in non-hematophagous species in this study. However, the limited number of individuals sampled per species precludes definitive conclusions about their role in virus maintenance. It is essential to continue sampling across species and regions to gain a clearer understanding of their epidemiological relevance. Additionally, environmental and ecological factors may also contribute to the spatial distribution of rabies prevalence observed in this study. Both San Lucas Ojitlán and Tlaxiaco are municipalities with substantial livestock presence and suitable environmental conditions (e.g., temperature, humidity, and roosting availability), which may support higher densities of *D. rotundus* and facilitate viral transmission. Also, rabies incidence in bats may fluctuate seasonally, with potential peaks that coincide with climatic or ecological changes, as observed in other parts of Latin America [34].

Beyond disease surveillance, the findings underscore the urgent need for targeted public health interventions, given the broader context of the reemergence of rabies in Mexico, as well as recently confirmed outbreaks in humans, as reported by the Mexican Ministry of Health and in recent literature [35,36]. These cases emphasize the ongoing threat rabies poses to public health and the importance of ongoing surveillance, particularly in wildlife reservoirs.

Misconceptions about bats—especially the assumption that all species transmit rabies—can lead to the indiscriminate destruction of colonies, including ecologically important non-hematophagous bats. Therefore, community-based education programs are critical. These should promote an accurate understanding of bat ecology, highlight their role in pollination and pest control, and address the specific risks posed by vampire bats. Livestock vaccination campaigns, implementation of animal housing at night, and the establishment of buffer zones to limit *D. rotundus* interactions with livestock should be integral components of control strategies [37,38].

Finally, this study reinforces the importance of active surveillance as a complement to traditional passive monitoring systems. While passive surveillance remains essential for detecting symptomatic animals, it tends to overlook asymptomatic viral carriers and early transmission foci. The inclusion of apparently healthy individuals in surveillance efforts provides a more accurate depiction of the virus’s ecology and may support earlier interventions in both wildlife and livestock populations.

## Figures and Tables

**Table 1:** Individuals captured per species during fieldwork for this study.

Family	Species	N° Specimens
Phyllostomidae	*Desmodus rotundus*	129 (3) *
	*Dermanura azteca*	21
	*Anoura geoffroyi*	5
	*Sturnira parvidens*	5
	*Artibeus jamaicensis*	4
	*Sturnira hondurensis*	4
	*Glossophaga soricina*	3
	*Macrotus waterhousii*	3
	*Leptonycteris yerbabuenae*	1
	*Dermanura phaeotis*	1
	*Dermanura watsoni*	1
Emballonuridae	*Balantiopteryx plicata*	12
Vespertilionidae	*Eptesicus fuscus*	3
	*Myotis* sp.	2

* In parentheses, positive cases of rabies by bat species.

## Data Availability

The original contributions presented in this study are included in the article. Further inquiries can be directed to the corresponding author.

## Abbreviations

The following abbreviations are used in this manuscript:

LESP	Laboratorio Estatal de Salud Pública
CPA LBS3s	Laboratorio de Bioseguridad Nivel 3 de la Comisión México-Estados Unidos para la Prevención de Fiebre Aftosa
LDPA Villahermosa	Laboratorio De Patología Animal Villahermosa
CENAPRECE	Centro Nacional de Programas Preventivos y Control de Enfermedades
SENASICA	Servicio Nacional de Sanidad, Inocuidad y Calidad Agroalimentaria
CENASA	Centro Nacional de Servicios de Diagnóstico en Salud Animal
DIF	Direct Immunofluorescence
WHOA	World Organization for Animal Health
SAGARPA	Secretaría de Agricultura, Ganadería, Desarrollo Rural, Pesca y Alimentación
OMS	Organización Mundial de la Salud
CEFPPO	Comité Estatal para el Fomento y Protección Pecuaria del Estado de Oaxaca
SINAVE	Sistema Nacional de Vigilancia Epidemiológica
SIRVERA	Sistema de Información Regional para la Vigilancia Epidemiológica de la Rabia

## Appendix A

### Appendix A.1

**Table A1:** Historical record of rabies cases in Oaxaca.

Year	Municipality	Physiographic Province	Species	Laboratory
2007	San Pedro Mixtepec	Sierra Madre del Sur	Canine	CENAPRECE
2007	Pinotepa Nacional	Pacific Coastal Plain	Canine	CENAPRECE
2007	Pinotepa Nacional	Pacific Coastal Plain	Canine	CENAPRECE
2007	Pinotepa Nacional	Pacific Coastal Plain	Canine	CENAPRECE
2007	San Vicente Coatlán	Valles Centrales	Human	CENAPRECE
2008	Pinotepa Nacional	Pacific Coastal Plain	Canine	CENAPRECE
2014	Santiago Jocotepec	Sierra Madre de Oaxaca	Bovine	LESP Oaxaca
2014	San Juan Cotzocon	Sierra Madre de Oaxaca	Bovine	LESP Oaxaca
2015	Santiago Jocotepec	Sierra Madre de Oaxaca	Bovine	CPA LBS3
2015	Constancia del Rosario	Sierra Madre del Sur	Bovine	CPA LBS3
2015	Heroica Ciudad de Tlaxiaco	Western Mountains and Valleys	Ovine	CPA LBS3
2015	Matias Romero Avendaño	Isthmus Depression of Tehuantepec	Bovine	LESP Oaxaca
2015	Matias Romero Avendaño	Isthmus Depression of Tehuantepec	Bovine	LESP Oaxaca
2015	Santiago Minas	Western Mountains and Valleys	Hematophagous bat	LESP Oaxaca
2015	Santiago Minas	Western Mountains and Valleys	Hematophagous bat	LESP Oaxaca
2015	San Pedro Mixtepec	Sierra Madre del Sur	Bovine	CPA LBS3
2016	San Pedro Tapanatepec	Tehuantepec Coastal Plain	Bovine	LESP Oaxaca
2016	San Juan Bautista Tuxtepec	Gulf Coastal Plain	Bovine	LESP Oaxaca
2016	Matias Romero Avendaño	Isthmus Depression of Tehuantepec	Bovine	LDPA Villahermosa
2016	Matias Romero Avendaño	Isthmus Depression of Tehuantepec	Bovine	LDPA Villahermosa
2016	Santa Maria Tlahuitoltepec	Sierra Madre de Oaxaca	Bovine	LESP Oaxaca
2016	Heroica Ciudad de Tlaxiaco	Western Mountains and Valleys	Ovino	LESP Oaxaca
2016	Santa Maria Tlahuitoltepec	Sierra Madre de Oaxaca	Hematophagous bat	LESP Oaxaca
2016	Heroica Ciudad de Tlaxiaco	Western Mountains and Valleys	Hematophagous bat	LESP Oaxaca
2016	San Agustin de Las Juntas	Central Mountains and Valleys	Bovine	CPA LBS3
2016	Mesones Hidalgo	Pacific Coastal Plain	Bovine	LESP Oaxaca
2016	Pinotepa Nacional	Pacific Coastal Plain	Bovine	LESP Oaxaca
2016	Heroica Ciudad de Tlaxiaco	Western Mountains and Valleys	Bovine	LESP Oaxaca
2016	Heroica Ciudad de Tlaxiaco	Western Mountains and Valleys	Bovine	LESP Oaxaca
2016	Heroica Ciudad de Tlaxiaco	Western Mountains and Valleys	Bovine	LESP Oaxaca
2016	San Pedro Amuzgos	Pacific Coastal Plain	Bovine	LESP Oaxaca
2017	San Juan Cacahuatepec	Pacific Coastal Plain	Bovine	LESP Oaxaca
2017	Loma Bonita	Gulf Coastal Plain	Bovine	LESP Oaxaca
2017	Santiago Yolomecatl	Western Mountains and Valleys	Bovine	CENASA
2018	San Juan Evangelista Analco	Sierra Madre de Oaxaca	Bovine	CENASA
2018	San Juan Bautista Tuxtepec	Gulf Coastal Plain	Bovine	CENASA
2018	Putla Villa de Guerrero	Sierra Madre del Sur	Bovine	CENASA
2018	San Juan Evangelista Analco	Sierra Madre de Oaxaca	Bovine	CENASA
2018	Santa Ana Yareni	Sierra Madre de Oaxaca	Bovine	CENASA
2019	San Francisco del Mar	Tehuantepec Coastal Plain	Bovine	LESP Oaxaca
2019	San Blas Atempa	Tehuantepec Coastal Plain	Bovine	LESP Oaxaca
2019	Santo Domingo Zanatepec	Tehuantepec Coastal Plain	Bovine	LESP Oaxaca
2019	Santo Domingo Zanatepec	Tehuantepec Coastal Plain	Bovine	LESP Oaxaca
2019	Santo Domingo Teojomulco	Western Mountains and Valleys	Bovine	CENASA
2019	Reforma de Pineda	Tehuantepec Coastal Plain	Bovine	CENASA
2019	San Juan Bautista Tuxtepec	Gulf Coastal Plain	Bovine	LESP Oaxaca
2019	Santo Domingo Armenta	Pacific Coastal Plain	Bovine	LESP Oaxaca
2019	Loma Bonita	Gulf Coastal Plain	Bovine	LESP Oaxaca
2019	San Pedro Comitancillo	Tehuantepec Coastal Plain	Equine	CPA LBS3
2019	San Francisco Ixhuatan	Tehuantepec Coastal Plain	Equine	CPA LBS3
2019	San Juan Bautista Tuxtepec	Gulf Coastal Plain	Bovine	CPA LBS3
2019	Reforma de Pineda	Tehuantepec Coastal Plain	Bovine	LESP Oaxaca
2019	San Pedro Tapanatepec	Tehuantepec Coastal Plain	Bovine	LESP Oaxaca
2019	San Juan Bautista Tuxtepec	Gulf Coastal Plain	Bovine	LESP Oaxaca
2020	San Miguel Chimalapa	Sierra Madre del Sur de Oaxaca y Chiapas	Bovine	LESP Oaxaca
2020	San Juan Cotzocon	Sierra Madre de Oaxaca	Bovine	LDPA Villahermosa
2020	San Juan Bautista Tuxtepec	Gulf Coastal Plain	Bovine	LESP Oaxaca
2020	San Jose Chiltepec	Gulf Coastal Plain	Equine	CPA LBS3
2020	Santo Domingo Teojomulco	Western Mountains and Valleys	Bovine	LESP Oaxaca
2020	San Juan Evangelista Analco	Sierra Madre de Oaxaca	Hematophagous bat	LESP Oaxaca
2020	San Juan Evangelista Analco	Sierra Madre de Oaxaca	Hematophagous bat	LESP Oaxaca
2020	Matias Romero Avendaño	Isthmus Depression of Tehuantepec	Bovine	LESP Oaxaca
2021	Santiago Yaveo	Gulf Coastal Plain	Bovine	LESP Oaxaca
2021	Asuncion Ixtaltepec	Tehuantepec Coastal Plain	Bovine	LESP Oaxaca
2021	San Juan Cotzocon	Sierra Madre de Oaxaca	Bovine	LESP Oaxaca
2021	San Lorenzo	Gulf Coastal Plain	Bovine	LESP Oaxaca
2021	San Juan Bautista Tuxtepec	Gulf Coastal Plain	Bovine	LESP Oaxaca
2021	San Juan Bautista Tuxtepec	Gulf Coastal Plain	Bovine	LESP Oaxaca
2021	Huajuapan de Leon	Western Mountains and Valleys	Bovine	CPA LBS3
2022	Magdalena Tequisistlan	Central Mountains and Valleys	Bovine	LESP Oaxaca
2022	Magdalena Tequisistlan	Central Mountains and Valleys	Bovine	LESP Oaxaca
2022	San Lorenzo	Western Mountains and Valleys	Human	LESP Oaxaca
2022	San Lorenzo	Western Mountains and Valleys	Human	LESP Oaxaca
2022	Santiago Choapam	Sierra Madre de Oaxaca	Bovine	LESP Oaxaca
2022	Heroica Ciudad de Tlaxiaco	Western Mountains and Valleys	Bovine	LESP Oaxaca
2022	Heroica Ciudad de Tlaxiaco	Western Mountains and Valleys	Bovine	LESP Oaxaca
2022	Heroica Ciudad de Tlaxiaco	Western Mountains and Valleys	Bovine	LESP Oaxaca
2022	Santa Maria Petapa	Sierra Madre de Oaxaca	Beefalo	LESP Oaxaca
2022	San Juan Lalana	Sierra Madre de Oaxaca	Bovine	LESP Oaxaca
2022	Matias Romero Avendaño	Isthmus Depression of Tehuantepec	Bovine	LESP Oaxaca
2022	San Pedro Mixtepec	Sierra Madre del Sur	Equine	CENASA
2022	Jalapa de Diaz	Gulf Coastal Plain	Bovine	LESP Hidalgo
2022	San Gabriel Mixtepec	Sierra Madre del Sur	Bovine	LESP Oaxaca
2022	San Lorenzo Texmelucan	Sierra Madre del Sur	Human	SIRVERA
2022	San Lorenzo Texmelucan	Sierra Madre del Sur	Human	SIRVERA
2023	San Juan Lalana	Sierra Madre de Oaxaca	Bovine	LESP Oaxaca
2023	Loma Bonita	Gulf Coastal Plain	Bovine	LESP Oaxaca
2023	San Lucas Ojitlan	Gulf Coastal Plain	Equine	CENASA
2023	Loma Bonita	Gulf Coastal Plain	Bovine	LESP Oaxaca
2023	San Juan Lalana	Sierra Madre de Oaxaca	Bovine	LESP Oaxaca
2023	San Juan Lalana	Sierra Madre de Oaxaca	Bovine	LESP Oaxaca
2023	Santa Maria Zacatepec	Pacific Coastal Plain	Bovine	LESP Oaxaca
2023	Tututepec de Melchor Ocampo	Pacific Coastal Plain	Bovine	LESP Oaxaca
2023	Santa Maria Petapa	Sierra Madre de Oaxaca	Bovine	LESP Oaxaca
2024	Santa Maria Yucuiti	Western Mountains and Valleys	Human	SINAVE
2024	Santa María Tonameca	Sierra Madre del Sur	Human	SINAVE

### Appendix A.2

**Table A2:** Bat species on which IDF was performed to determine rabies in Oaxaca. Numbers in parentheses indicate positive cases recorded by species and location.

Scientific Name	Location	Specimens
*Desmodus rotundus*	San Miguel Cuevas, Santiago Juxtlahuaca, Western Mountains and Valleys	11
*Anoura geoffroyi*		1
*Leptonycteris yerbabuenae*		1
*Eptesicus fuscus*		3
*Myotis* sp.		2
*Dermanura azteca*		1
*Artibeus jamaicensis*	La Ventosa, Juchitán, Tehuantepec Coastal Plain	2
*Desmodus rotundus*	San Sebastián de las Grutas, Villa Sola de Vega, Western Mountains and Valleys	2
*Dermanura azteca*		5
*Artibeus jamaicensis*		2
*Anoura geoffroyi*		1
*Desmodus rotundus*	Santo Domingo Teojomulco, Sola de Vega, Western Mountains and	26
*Glossophaga soricina*	Valleys	2
*Anoura geoffroyi*		1
*Sturnira hondurensis*		1
*Desmodus rotundus*	Ayoquezco de Aldama, Central Valleys	15
*Desmodus rotundus*	Heroica Ciudad de Tlaxiaco, Western Mountains and Valleys	13 (1)
*Desmodus rotundus*	Santiago Apoala Nochixtlán, Western Mountains and Valleys	8
*Dermanura azteca*		15
*Anoura geoffroyi*		2
*Desmodus rotundus*	San Juan Evangelista Analco, Sierra Madre de Oaxaca	15
*Desmodus rotundus*	La Cañada, Santa Inés del Monte Zaachila, Central Valleys	2
*Desmodus rotundus*	La Soledad, Santa Inés del Monte Zaachila, Central Valleys	2
*Desmodus rotundus*	Santa María Mixtequilla, Sierra Madre del Sur	8
*Macrotus waterhousii*		3
*Balantiopteryx plicata*		12
*Desmodus rotundus*	Rancho 1 uvero, Palomares Matías Romero Juchitán, Isthmus Depression of Tehuantepec	3
*Desmodus rotundus*	Rancho 2, Palomares, Matías Romero Juchitán, Gulf Coastal Plain	7
*Sturnira parvidens*		4
*Sturnira hondurensis*		2
*Dermanura phaeotis*		1
*Dermanura watsoni*		1
*Desmodus rotundus*	San Miguel Chimalapas, Tehuantepec Coastal Plain	17
*Glossophaga soricina*	San Lucas Ojitlán, Tuxtepec, Gulf Coastal Plain	1
*Sturnira hondurensis*		1
*Desmodus rotundus*		4 (2)
*Desmodus rotundus*	Palo de lima, San Lorenzo Texmelucan Juquila, Western Mountains and	4
*Sturnira parvidens*	Valleys	1
*Desmodus rotundus*	El Carrizal, San Lorenzo Texmelucan Juquila, Western Mountains and Valleys	5
